# Epidermal biopolysaccharides from plant seeds enable biodegradable turbulent drag reduction

**DOI:** 10.1038/s41598-019-54521-3

**Published:** 2019-12-04

**Authors:** Anoop Rajappan, Gareth H. McKinley

**Affiliations:** Department of Mechanical Engineering, Massachusetts Institute of Technology, Cambridge, Massachusetts 02139 USA

**Keywords:** Mechanical engineering, Polymers, Rheology, Fluid dynamics

## Abstract

The high cost of synthetic polymers has been a key impediment limiting the widespread adoption of polymer drag reduction techniques in large-scale engineering applications, such as marine drag reduction. To address consumable cost constraints, we investigate the use of high molar mass biopolysaccharides, present in the mucilaginous epidermis of plant seeds, as inexpensive drag reducers in large Reynolds number turbulent flows. Specifically, we study the aqueous mucilage extracted from flax seeds (*Linum usitatissimum*) and compare its drag reduction efficacy to that of poly(ethylene oxide) or PEO, a common synthetic polymer widely used as a drag reducing agent in aqueous flows. Macromolecular and rheological characterisation confirm the presence of high molar mass (≥2 MDa) polysaccharides in the extracted mucilage, with an acidic fraction comprising negatively charged chains. Frictional drag measurements, performed inside a bespoke Taylor-Couette apparatus, show that the as-extracted mucilage has comparable drag reduction performance under turbulent flow conditions as aqueous PEO solutions, while concurrently offering advantages in terms of raw material cost, availability, and bio-compatibility. Our results indicate that plant-sourced mucilage can potentially serve as a cost-effective and eco-friendly substitute for synthetic drag reducing polymers in large scale turbulent flow applications.

## Introduction

The addition of small quantities of soluble, high molar mass polymer chains has long been known to yield large reductions in the frictional pressure drop and skin friction drag in turbulent pipe and boundary layer flows^1–8^. The most striking aspect of turbulent drag reduction by polymers is perhaps its efficacy; very sparse concentrations of high polymers, sometimes as low as 1–10 ppm, are sufficient to cause significant alterations in the near-wall turbulence dynamics, yielding appreciable reductions in wall shear stress and pumping head requirements of up to 20–40%^2,7^. At higher concentrations, peak drag reduction levels as high as 70–80% have been achieved^2^. The utility of polymer additives for the purposes of drag reduction and flow control has been demonstrated in a diverse array of engineering applications involving turbulent flows, including their use as flow enhancers in pipelines, sewers, district heating networks, field irrigation systems^9^, firefighting equipment^10^, and hydraulic fracturing fluids^11^, as frictional drag reducers for ships and underwater vehicles^2,12^, and even as intravenous haemorheological modifiers to inhibit atherosclerosis and to aid resuscitation from haemorrhagic shock^13–16^. An example of the successful deployment of drag-reducing polymers on a large scale is seen in the Trans-Alaska Pipeline System (TAPS), wherein the injection of polymer additives has been used since 1979 to enhance throughput in the long distance transport of crude oil^17^. Nature also provides examples of drag reduction by polymers; for instance, the epidermal mucus (slime) of several species of fish has been shown to promote boundary layer drag reduction at fast swimming speeds^18,19^.

The ejection of drag-reducing polymers into the boundary layer has been explored over the years as a prospective technique for the mitigation of hydrodynamic drag on marine and naval vessels^2,12^. Skin friction on the hull accounts for about 50% of the total drag on ships, and 60% of the drag on submarines^12^; an estimated 60% of the propulsive power of a typical ship is expended in overcoming this frictional resistance^20^. Methods of reducing skin friction, even moderately, thus offer the potential to generate substantial savings in fuel and operating costs, improve speed and propulsive efficiency, and reduce exhaust emissions into the atmosphere. Experiments conducted in towing tanks and water tunnel facilities have consistently shown that injecting a concentrated polymer stock solution into the near-wall region of a turbulent boundary layer can produce appreciable reductions in skin friction, sometimes as high as 75%^21^. In 1971, full scale drag reduction trials were performed on a 140-feet long coastal minesweeper, the HMS Highburton, during which a concentrated solution of 4 MDa polyethylene oxide (PEO) was ejected through slots in the hull to achieve a nominal polymer concentration of 10 ppm in the boundary layer^22^. Notwithstanding adverse weather conditions and the uneven mixing of the polymer in the boundary layer, a net reduction of 12.7% in the total drag, and a 17% decline in diesel consumption, were realised. Despite the considerable body of favourable experimental evidence, the deployment of polymers in real-life marine applications has been largely impeded by practical difficulties, primarily the material cost of synthetic high molar mass polymer additives; water-soluble polymers such as PEO are too expensive to be viable in commercial marine operations^2,12,23^. This, however, provides an opportunity where inexpensive plant-sourced biopolymers can play an impactful role, serving as equally efficacious yet cost-effective alternatives to synthetic drag-reducing agents.

A number of natural polymers have been explored in the literature as potential drag reducers, including water-soluble derivatives of cellulose, various food gums, starch (amylopectin), alginate, carrageenan, chitosan, DNA and even the pectinous sap of plants such as okra^24–26^. In particular, past investigations have focused extensively on two (relatively expensive) food gums, namely xanthan and guar, on account of their widespread use in drilling muds and as proppant suspenders in hydraulic fracturing fluids^11,27^. By contrast, the aqueous mucilage derived from the seeds of plants such as flax, chia or psyllium, despite being inexpensive sources of high molar mass polysaccharides^28–30^, has not been studied in any detail as potential drag reducing agents in turbulent flows. We have recently explored the rheology of these seed brans as liquid food modifiers and found that flax mucilage, in particular, can dramatically confer viscoelasticity to aqueous solutions^31^. Besides their low cost, the use of seed mucilage entails several practical advantages over synthetic drag reducing polymers; it is biodegradable^25,32,33^ and non-toxic to aquatic life^34^, essential requirements for additives intended for release into oceans and navigable water bodies. Furthermore, it can be easily eluted from raw, unprocessed seeds using simple hot water extraction techniques (for example, using low-grade waste heat from the engine or steam boilers), requiring no chemical synthesis or solvents in its production. Below, we report the experimental characterisation of the drag reducing properties of mucilage extracted from flaxseed, and compare its performance to that of aqueous polyethylene oxide (PEO), a synthetic polymer commonly employed as a drag reducer in aqueous flows. We also discuss briefly the impact of mechanical degradation and salinity on the drag reduction efficacy of mucilage polysaccharides, addressing the two exigent concerns involved in selecting polymer additives for turbulent internal flows and marine environments.

## Results

### Polysaccharide extraction and characterisation

**Figure 1:**
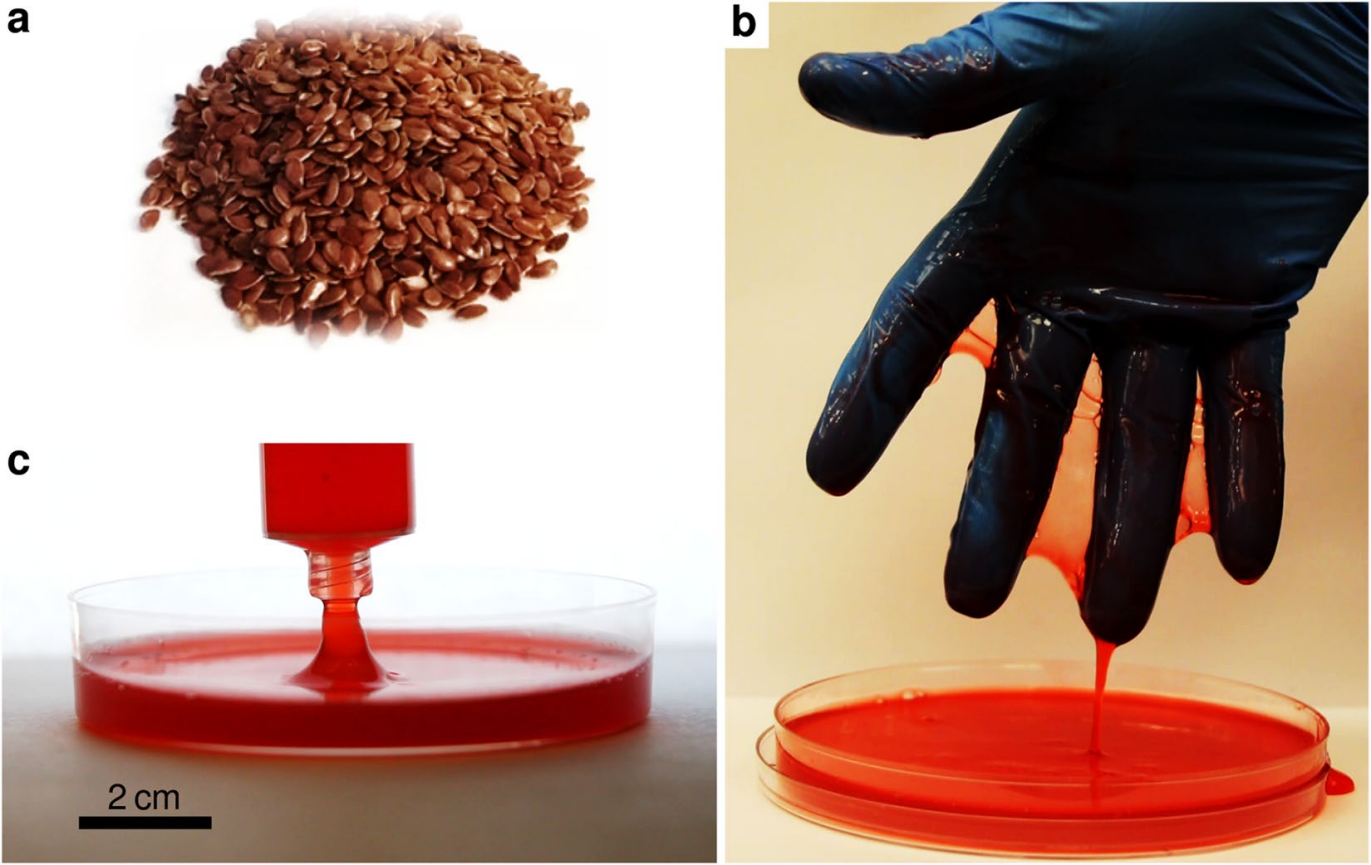
(**a**) Brown flaxseed (*Linum usitatissimum*) used in the extraction of mucilage. (**b**) As-extracted viscoelastic aqueous mucilage from flax seeds. A seed to water weight ratio of 1:8 was used, with a red food dye added to aid visualisation. (**c**) Open siphoning of mucilage, possible because of viscoelasticity imparted by dissolved high molar mass polysaccharides. The full movie is included in the Supplementary Material.

We extracted the water-soluble polysaccharide content, or *mucilage*, from the mucous epidermis (seed coat) of the brown variety of flax seeds (*Linum usitatissimum*, see Fig. 1a), by soaking unprocessed, whole seeds in hot deionised water at 80 °C for 30 min; this protocol has been previously reported as optimal for the aqueous extraction of mucilage with minimal protein contamination^35^. The principal component of the mucilage from flax seeds, constituting approximately 75% by weight, is known to be a high molar mass (≥10^6^ g/mol) neutral arabinoxylan, and the remainder mostly an acidic fraction consisting of various negatively charged rhamnogalacturonans of lower molar mass^28^. The as-extracted aqueous mucilage solution is moderately viscoelastic on account of these dissolved long-chain polysaccharides, and can be drawn up in a ‘tubeless siphon’^36^ due to the large increase in extensional viscosity imparted by stretched polymer chains (see Fig. 1b,c).

**Figure 2:**
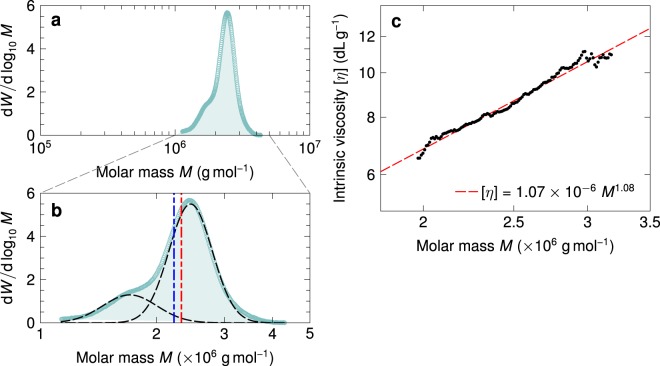
(**a**) Molar mass distribution of purified flax mucilage, determined using size exclusion chromatography. *W* is the cumulative mass fraction of the eluted polymer. (**b**) An enlarged view of the chromatogram shows that the narrow peak in (**a**) is bimodal, and can be resolved into two distinct log-normal distributions represented by the black dashed curves. The red and blue vertical lines denote, respectively, the weight averaged molar mass *M*_*w*_ = 2.32 × 10^6^ g/mol and the number averaged molar mass *M*_*n*_ = 2.22 × 10^6^ g/mol of the aggregate distribution. (**c**) The intrinsic viscosity [*η*] of mucilage fractions as a function of the molar mass *M*. The red dashed line is a linear least-squares fit used to estimate the Mark-Houwink parameters *K* and *a*.

For molecular characterisation of mucilage polysaccharides, we employed a 1:25 weight ratio of flax seeds to water during the extraction stage. The aqueous mucilage solution so obtained, after filtration, contained dissolved constituents amounting to roughly 9.8% of the original dry weight of seeds used. Of this, about 4% was a gel-like fraction separable by centrifugation, and approximately 40% consisted of low molar mass solutes below 100 kDa. After removing these components, we analysed the remaining high molar mass polysaccharide fraction using triple-detection size exclusion chromatography (SEC). The resulting molar mass distribution of polysaccharides is shown in Fig. 2a. Although the molar mass of the sample is narrowly distributed and relatively monodisperse, on closer inspection the distribution is discernibly bimodal (as seen in Fig. 2b), and is well described by a superposition of two log normal peaks, centred at *M*_1_ = 1.71 × 10^6^ g/mol and *M*_2_ = 2.45 × 10^6^ g/mol respectively. Overall, this yields a weight averaged molar mass of *M*_*w*_ = 2.32 × 10^6^ g/mol for the aggregate sample, and a polydispersity index PDI = 1.05.

Figure 2c shows the intrinsic viscosity [*η*], obtained from the SEC inline differential viscosity detector, as a function of the chain molar mass *M*, determined using dual-angle light scattering. By performing a linear least-squares fit of the Mark-Houwink-Sakurada equation^37^ [*η*] = *KM*^*a*^ to the data, we estimated the Mark-Houwink parameters for the flax polysaccharides to be *a* = 1.08 ± 0.02 and *K* = (1.07 ± 0.27) × 10^−6^ dL/g (g/mol)^−a^. We note, however, that these estimates may have large associated uncertainties arising from the limited range of molar masses available for fitting, on account of the narrow polydispersity of the original sample. Furthermore, the values of *K* and *a* obtained above correspond to a solvent ionic strength of 0.1 M, equivalent to the molarity of the aqueous NaNO_3_ solution employed as the eluent during SEC analysis. A value of *a* = 1.08 for the Mark-Houwink exponent yields a Flory exponent of *ν* = (*a* + 1)/3 = 0.69, larger than the value of *ν* = 0.6 expected for flexible chains in good solvents^37^. This suggests an expanded conformation for the charged polysaccharide constituents in the mucilage, which is further evidenced by the results of our viscometric study to be discussed below.

We also measured the intrinsic viscosity [*η*] of aqueous mucilage independently using a suspended-level capillary viscometer, following the conventional procedure of extrapolating the reduced viscosity and the inherent viscosity concurrently to zero concentration^38^. The reduced viscosity of desalted flax mucilage in deionised water was observed to initially increase with decreasing concentration, in accordance with the Fuoss equation^39,40^ (details provided in the Supplementary Material). This atypical behaviour in dilution is a well-known attribute of polyelectrolytes, and is ascribed primarily to chain expansion resulting from the increased dissociation of ionisable groups, and the concomitant increase in electrostatic intramolecular repulsion^41^. The reduced viscosity increases as the polyelectrolyte chains progressively adopt a stretched conformation, and eventually attains a local maximum when the chains reach maximal expansion at very low concentrations. Further dilution results in a decrease in reduced viscosity, as in the case of uncharged polymer chains^41^. In the case of flax mucilage, we observed this transition at a concentration of approximately *c* = 46 mg/L; beyond this point, the usual Huggins extrapolation in the linear region yielded an intrinsic viscosity of [*η*] = 107.5 dL/g at 25 °C, which is an order of magnitude larger than the intrinsic viscosity of neutral, flexible coils of similar size (for comparison, PEO with a molar mass of 2 × 10^6^ g/mol has an intrinsic viscosity of [*η*]_PEO_ = 9.13 dL/g in water), again suggesting the presence of extended rod-like chains in the mucilage. This polyelectrolyte-like behaviour of aqueous flax mucilage is consistent with the presence of an acidic fraction comprising charged, semiflexible rhamnogalacturonan chains, as has been reported in previous studies^28^.

Furthermore, the intrinsic viscosity of mucilage was observed to be highly sensitive to the ionic strength of the solution, falling by more than an order of magnitude from [*η*] = 107.5 dL/g at zero salt concentration, to [*η*] = 8.57 dL/g in a 0.5 M aqueous NaCl solution^42,43^. This can be explained as a consequence of effective charge screening by the added ions, permitting the expanded chains to revert to a random coil configuration^44^. Insofar as we intend to use the crude seed extract directly for drag reduction, without any subsequent purification or desalting, it was pertinent to characterise the properties of the mucilage at ionic strengths similar to that of the original, as-extracted solution. To this end, we repeated the intrinsic viscosity measurements using an isoionic dilution procedure (described in Methods), using the low molar mass fraction isolated from the extract itself as the diluent. No discernible polyelectrolyte-like effect was observed during experiments at this ionic strength^42^, and an isoionic intrinsic viscosity of [*η*]_FM_ = 10.03 dL/g at 25 °C was obtained, which is quite close to that of aqueous polyethylene oxide (PEO) of comparable molar mass (*M*_*w*_ = 2 × 10^6^ g/mol, [*η*]_PEO_ = 9.13 dL/g). On account of this close correspondence in terms of the molar mass and intrinsic viscosity (and thereby, the chain size in solution), we henceforth adopt 2 MDa PEO as a benchmark against which to compare the drag reduction performance of flax mucilage as a bio-sourced alternative. A further motivation for our choice is that PEO is among the synthetic polymers most commonly employed for drag reduction in aqueous flows, and 2 MDa PEO has been successfully used as a drag-reducing polymer in previous studies^21,45^. A comparison of the drag reduction performance of flax mucilage with a dilute PEO solution of higher molar mass (*M*_*w*_ = 5 × 10^6^ g/mol, *c* = 10 ppm, *c*[*η*] = 0.02) is also included in the Supplementary Material.

For turbulent drag reduction measurements (described in detail in the next section), we employed dilute mucilage extracts prepared at varying seed-to-water ratios ranging from 1:100 to 1:800 by weight, yielding solutions with different concentrations *c* of dissolved polysaccharides. After extraction, the supernatant solution was filtered once through a coarse (grade 90) cotton mesh to remove gross particulates, but was otherwise used as is in flow experiments without any further purification or processing. To ensure a fair comparison at the same “effective” polymer concentration, we performed flow tests using aqueous PEO solutions having identical values of the normalised concentration *c*[*η*] as the mucilage extracts. The flax mucilage extracts used for drag reduction measurements in our study had polymer concentrations in the range of 0.26 ≤ *c*[*η*] ≤ 1.3, therefore being largely in the dilute (or weakly semi-dilute) regime. The chain overlap concentration c* may be estimated approximately as c^*^ = 1/[*η*]^37^, yielding the values (c*)_FM_ = 1.00 g/L for flax mucilage, and (c*)_PEO_ = 1.10 g/L for aqueous 2 MDa PEO.

**Figure 3:**
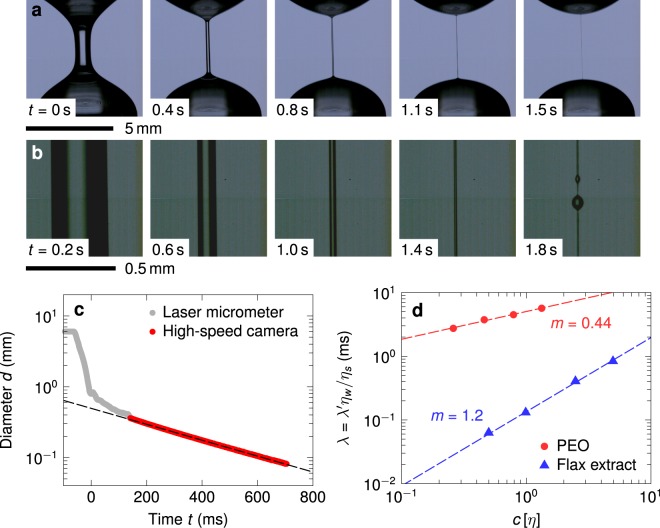
(**a**) Elastocapillary thinning of PEO solution (*c*[*η*] = 1.33) in 0.7 g/mL aqueous sucrose (solvent viscosity *η*_*s*_ = 0.019 Pa s) on a CaBER device. The legend indicates the elapsed time, with *t* = 0 corresponding to the instant when the plates come to rest after separation. (**b**) Magnified images of the filament midpoint recorded using a high speed camera, and used to obtain the filament diameter *d* as a function of time. The final image shows the development of beads-on-a-string (BoaS) instability. (**c**) Evolution of the filament diameter *d*(*t*) with time *t*. The diameter at initial times was measured using a laser micrometer, and at later times was estimated from the high speed recording (**b**) shown above. The dashed line is a least-squares fit of the form *d*(*t*) ~ exp (−*t*/3*λ*′) to the data in the exponential thinning region. (**d**) Estimated values of the extensional relaxation time *λ* for aqueous PEO and flax mucilage solutions, plotted against the normalised polymer concentration *c*[*η*]. The relaxation times *λ* in water were obtained by rescaling the relaxation times *λ*′ in aqueous sucrose by the ratio of solvent viscosities. Error bars are smaller than or comparable to the size of the data markers, and are omitted for clarity.

Finally, as a means of characterising the rheological properties of mucilage, we used a capillary breakup extensional rheometer (CaBER) to estimate the extensional relaxation time *λ* of the polysaccharide chains in solution, at low concentrations relevant to drag reduction applications. In a typical CaBER experiment, a small volume of the test solution is confined between two coaxial end plates, and subjected to an axial step strain via a rapid separation of the plates to a fixed final gap. This induces a capillary-driven instability in the slender liquid bridge that forms between the plates. In the case of viscoelastic liquids, this eventually leads to the formation of a cylindrical, self-thinning liquid filament (see Fig. 3a,b) in which the capillary forces driving the uniaxial, purely extensional draining flow are balanced by the non-linear growth in elastic stresses arising from the progressive elongation and unravelling of dissolved polymer chains^46,47^. In this elasto-capillary thinning regime, the filament diameter *d*(*t*) decreases exponentially with time *t* according to

1 $$d(t)=d(0)\,\exp \,(\,-\,t/3\lambda ).$$

The evolution of the diameter *d*(*t*) is tracked experimentally using either a laser micrometer or a high-speed camera, and a least-squares fit of the data to Eq. 1 in the exponential thinning region, as shown in Fig. 3c, yields the extensional relaxation time *λ* of the polymer chains in solution^46–50^.

A simple theoretical estimate of the longest relaxation time of polymer chains in dilute solution is given by

2 $${\lambda }_{Z}\simeq \frac{[\eta ]{M}_{w}{\eta }_{s}}{RT}$$

which, excepting a numerical prefactor of order unity that depends on the solvent quality, is equivalent to the Zimm relaxation time of dilute Gaussian coils in the ‘non-draining’ limit of dominant intrachain hydrodynamic interactions^37^. In Eq. 2, *η*_*s*_ is the solvent viscosity, *T* the solution temperature, and *R* the universal gas constant. For the dilute solutions used in our drag reduction studies (0.26 ≤ *c*[*η*] ≤ 1.3), this gives an estimated relaxation time of (*λ*_*Z*_)_PEO_ = 0.66 ms and (*λ*_*Z*_)_FM_ = 0.84 ms for PEO and mucilage polysaccharides respectively, at 25 °C with pure water as the solvent. Obtaining accurate measurements of the chain relaxation time using CaBER becomes increasingly difficult for relaxation times approaching *λ* ≤ 1 ms, at which point the filament lifetime becomes so short that the elasto-capillary regime, described by Eq. 1, is experimentally inaccessible^51^. In addition, at the dilute concentrations mentioned above, the zero shear rate viscosity of aqueous polymer solutions are so low (*η*_0_ ≤ 3 mPa s) that inertial oscillations of the upper and lower liquid bulbs, triggered by the moving plates, persist throughout the observation window, vitiating diameter measurements and forcing premature breakage of the filament^51^. To surmount these practical difficulties, we added sucrose as a viscosifier to enhance the solvent viscosity *η*_*s*_ of the test solutions prior to CaBER measurements, thereby delaying filament rupture sufficiently to permit reliable measurements of the filament diameter *d*(*t*) in the elasto-capillary regime. From Eq. 2, the relaxation time increases linearly with solvent viscosity as *λ* ~ *η*_*s*_, a general result of bead-spring models that is true of flexible coils, semiflexible chains, and extended rigid rods in solution^37^. Rescaling the experimentally obtained relaxation times *λ*′ (measured in the aqueous sucrose solution) to account for the increased solvent viscosity, we thus obtained better-resolved estimates of the relaxation time *λ* = *η*_*w*_*λ*′/*η*_*s*_ of polymer chains in the original aqueous solution, *η*_*w*_ being the viscosity of pure water, and *η*_*s*_ that of the sucrose solution of higher viscosity employed as the solvent during CaBER measurements.

Figure 3d shows the variation in the extensional relaxation time *λ* for PEO and mucilage polysaccharides in water, as a function of the normalised concentration *c*[*η*]. The relaxation times *λ* are larger than the theoretical estimate *λ*_*Z*_ above by approximately an order of magnitude in the case of PEO, whereas they are comparable or smaller than *λ*_*Z*_ in the case of flax mucilage. The observation of an enhanced relaxation time for flexible chains such as PEO in uniaxial extensional flows has been consistently reported in numerous studies in the literature^48,52^. Equation 2, which is strictly valid only in the limit of infinite dilution, predicts a relaxation time *λ*_*Z*_ that is independent of the concentration of chains in solution. However, in strong extensional flows, the pervaded volume of the chain increases substantially as it undergoes a coil-stretch transition, resulting in interchain hydrodynamic interactions, and rendering the solution effectively semi-dilute^49,52^. This, in turn, manifests as a concentration dependence of the extensional relaxation time *λ* which persists down to concentrations far below c*, the usual threshold for single chain dynamics in weak shear flows. On the other hand, the addition of sucrose is known to lower the dielectric constant of aqueous solutions much like ionic electrolytes^53^, and the resulting charge screening can cause expanded polyelectrolyte chains to progressively revert to a random coil configuration. A decrease in solvent quality and radius of gyration in the presence of sucrose has indeed been reported for a number of polysaccharides in the literature^54–56^. A similar decrease in the intrinsic viscosity of mucilage, effected by the dissolved sucrose, could explain the smaller value of experimental relaxation times *λ*, in comparison to the theoretical value *λ*_*Z*_ estimated for isoionic conditions.

From Fig. 3d, we see that the relaxation time *λ* for both PEO and flax mucilage solutions exhibit a power-law behaviour of the form *λ* ~ (*c*[*η*])^*m*^, with exponents *m*_PEO_ = 0.44 and *m*_FM_ = 1.2 respectively. For unentangled, semi-dilute solutions of flexible chains, “blob” theories predict a scaling exponent *m* = (2 − 3*ν*)/(3*ν* − 1)^48,52^. Substituting *m*_PEO_ = 0.44 in this expression yields a Flory exponent of *ν* = 0.56 for aqueous PEO, which is close to, but slightly smaller than, the value of *ν* = 0.59 reported in the literature for dilute solutions of PEO in water^57^; this may be attributed to a decrease in solvent quality in the presence of sucrose. For flax mucilage, *m*_FM_ = 1.2 yields *ν* = 0.48, again indicative of strong charge screening and poor solvent quality of the sucrose solution.

### Turbulent drag reduction measurements

**Figure 4:**
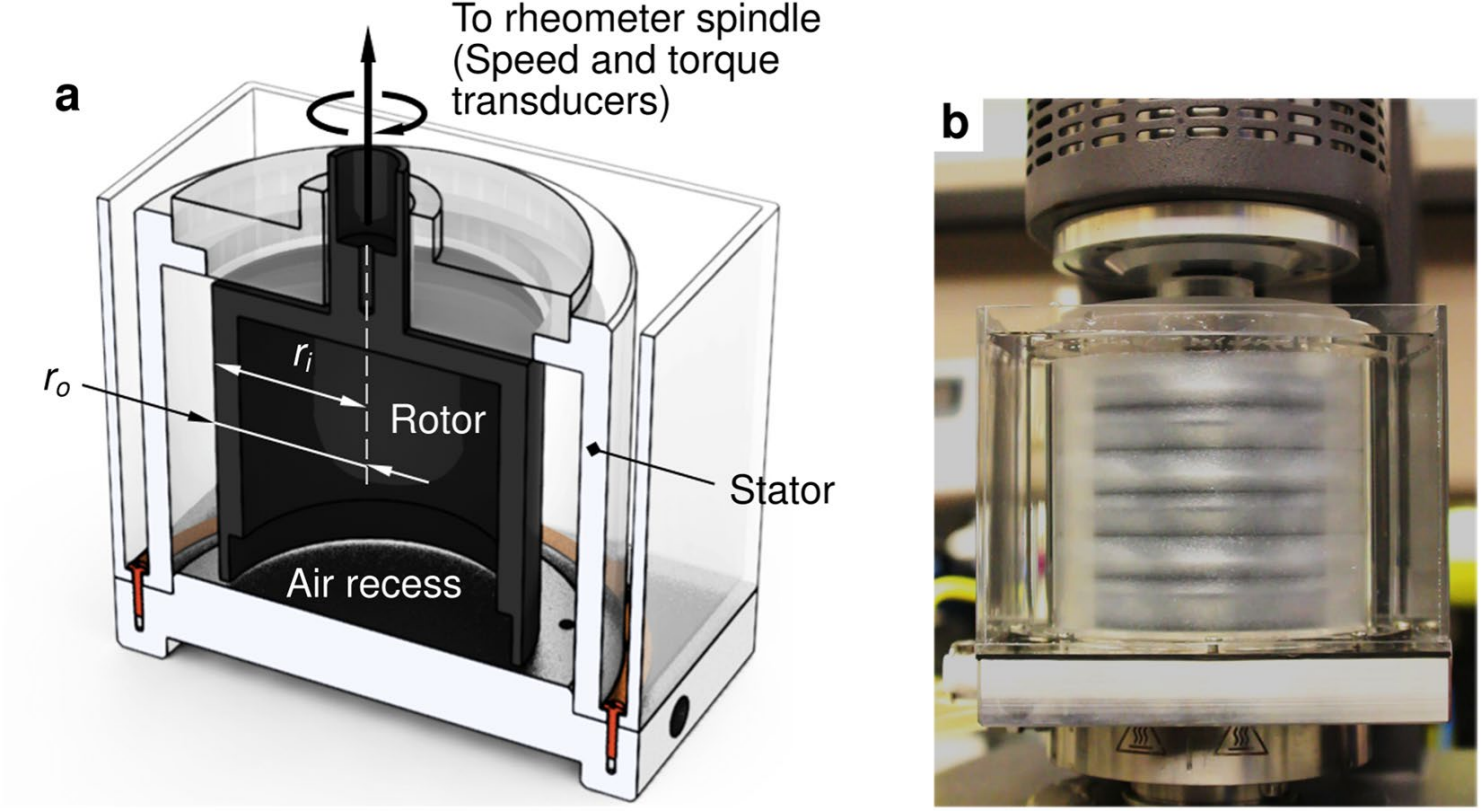
The bespoke Taylor-Couette (TC) apparatus used for flow measurements. (**a**) Schematic sectional view of the TC apparatus, showing the transparent stator and the end-recessed rotor. (**b**) The TC fixture mounted on the AR-G2 rheometer. Three pairs of counter-rotating Taylor vortices are visible at Re = 156, on filling the cell gap with a rheoscopic fluid consisting of synthetic mica flakes dispersed in a glycerol-water mixture. (Reproduced from A. Rajappan *et al*., Influence of textural statistics on drag reduction by scalable, randomly rough superhydrophobic surfaces in turbulent flow, Phys. Fluids **31**, 042107 (2019), with the permission of AIP Publishing).

We characterised the drag reduction efficacy of aqueous PEO and flax mucilage in fully turbulent shear flow generated inside the annular gap of a bespoke Taylor-Couette (TC) apparatus, depicted schematically in Fig. 4a. The rotating inner cylinder (rotor) has radius *r*_*i*_ = 38.1 mm and height *h* = 76.2 mm, and is enclosed by a stationary coaxial outer cylinder (stator) of radius *r*_*o*_ = 50.8 mm, forming a cylindrical annulus of radial width *w* = *r*_*o*_ − *r*_*i*_ = 12.7 mm in which the working fluid is contained; the TC fixture thus has a radius ratio *ζ* = *r*_*i*_/*r*_*o*_ = 0.75, and a gap aspect ratio *β* = *h*/*w* = 6.0. The rotor is coupled directly to, and is driven by, a commercial controlled-stress rotational rheometer (as seen in Fig. 4b), enabling precise measurements of its angular speed Ω, and the frictional torque ${\mathscr T}$ exerted by the fluid on its lateral cylindrical surface. The hollow recess within the rotor body remains air-filled during experiments, providing a shear-free interface at the bottom which effectively eliminates the extraneous torque from fluid friction that would otherwise act on the lower face.

In accordance with the usual convention for TC flows, we define the Reynolds number Re = *ρ*(*r*_*i*_Ω)*w*/*η* based on the gap width *w*, where *ρ* and *η* are, respectively, the density and dynamic viscosity of the working fluid. In the aforementioned configuration involving a fixed outer cylinder and a rotating inner cylinder, the primary (azimuthal) laminar flow in the TC apparatus is unstable above a critical rotation rate, and develops multiple pairs of counter-rotating Taylor vortices as the rotor speed Ω is increased^58^. Additional flow instabilities appear at higher speeds, generating a series of secondary flow patterns that progresses through wavy and turbulent vortex states^58^. At even larger rotation rates, these vortical structures break up and disappear completely, giving rise to featureless, fully turbulent shear flow in the annular gap when a critical value of the Reynolds number, Re = Re_*c*_, is exceeded^59,60^. In the particular case of our bespoke TC apparatus, this transition to featureless (Newtonian) turbulence was observed to occur at Re_*c*_ ≃ 11,000, as determined from the scaling of the baseline torque, measured in pure water, with the rotor speed (details included in the Supplementary Material).

**Figure 5:**
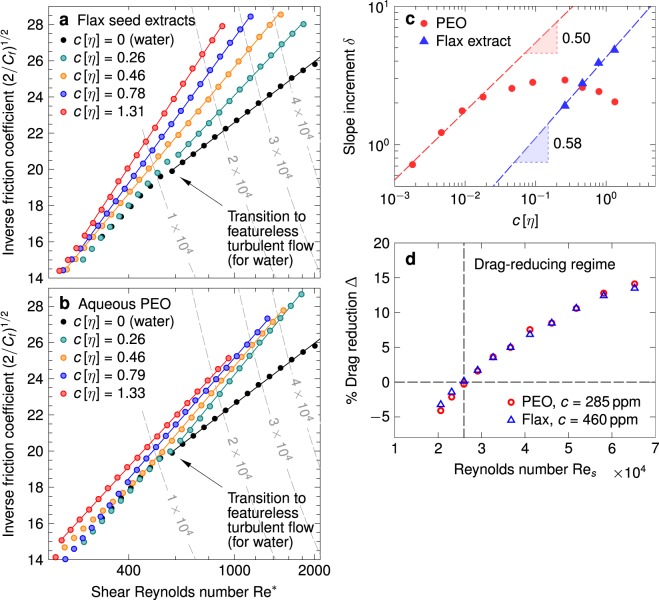
Experimental skin friction curves for (**a**) flax mucilage solutions and (**b**) aqueous PEO solutions at identical values of the normalised concentration *c*[*η*], plotted in Prandtl-von Kármán coordinates. The solid lines in both panels are linear least squares fits of Eq. 4 to the experimental data in the fully turbulent flow regime, and the dashed gray curves denote contours of constant Reynolds number Re. (**c**) The slope increment *δ* plotted as a function of the normalised concentration *c*[*η*] in log-log axes. The dashed lines are linear least squares fits to the data. (**d**) The percentage drag reduction Δ as a function of the Reynolds number Re for 285 ppm aqueous PEO, and 1:400 flax seed extract (*c* = 460 ppm). The two solutions show near identical performance in the drag-reducing regime (Re_*s*_ ≥ 26,000).

During a typical flow experiment, the angular speed Ω of the rotor was increased in discrete steps, and the resulting steady state frictional torque ${\mathscr T}$ was recorded. The average wall shear stress $\tau ={\mathscr T}/2\pi {r}_{i}^{2}h$ at the rotor surface, and the non-dimensional coefficient of friction *C*_*f*_ = 2*τ*/*ρ*(*r*_*i*_Ω)^2^, were then calculated from the experimentally measured torque versus speed curve. When the fluid inside the gap is Newtonian, one can use matched asymptotic expansions for the azimuthal angular momentum to show that the coefficient of friction *C*_*f*_ in fully turbulent Taylor-Couette flow (for Re > Re_*c*_) obeys a logarithmic friction law of the form^61^

3 $$\sqrt{\frac{2}{{C}_{f}}}=M\,\log \,{{\rm{Re}}}^{\ast }+N,$$

where Re* = Re(*C*_*f*_/2)^1/2^ = *ρ*(*τ*/*ρ*)^1/2^*w*/*η* is the shear Reynolds number at the inner wall, and the constants *M* and *N* are independent of the flow speed and fluid properties. Equation 3 is identical in form to the familiar Prandtl-Kármán friction law for turbulent pipe flows, although the values of *M* and *N* in this case are not universal, but are functions of the radius ratio *ζ* of the TC geometry^59,61^. In Fig. 5a,b, experimental data from our flow measurements are presented in the form of Prandtl-von Kármán plots, in which (2/*C*_*f*_)^1/2^ (the quantity on the left hand side of Eq. 3) is plotted against Re* on semi-logarithmic axes. The black data points denote baseline measurements in pure water, and a sharp change in the slope of this curve marks the transition to featureless Newtonian turbulence at Re = Re_*c*_. The friction data beyond this point showed good conformance to the functional form predicted by Eq. 3, and we used a linear least-squares fit in this region to extract the slope *M* of the (Newtonian) baseline.

In the case of aqueous PEO and flax mucilage solutions, the transition to elastic turbulence, in general, was observed to occur at lower flow speeds in comparison to the pure Newtonian solvent^62^. Given that solutions of long chain polymers, at the large localised strain rates encountered in turbulence, may concurrently undergo both viscous shear-thinning in regions of predominantly shear flow as well as strain hardening in regions of strong extensional flow, there exists some degree of incertitude in the correct choice of the solution viscosity *η* to be used in the definition of Re*; different authors have, in the past, set it equal to the solvent viscosity *η*_*s*_^4^, the solution viscosity *η*_0_ at zero shear rate^63^, the viscosity *η*_∞_ at infinite shear rate (obtained from a Carreau-Yasuda fit)^62^, or the local value of the shear viscosity $\eta (\dot{\gamma })$ corresponding to the shear rate $\dot{\gamma }$ at the wall^64^. For the data in Fig. 5a,b, we have opted to use the zero shear rate viscosity *η*_0_. The dilute solutions used in our experiments have zero shear rate viscosities not too different from that of the pure solvent, and showed no appreciable shear thinning at shear rates $\dot{\gamma }\le {10}^{3}\,{{\rm{s}}}^{-1}$ (see Supplementary Material). More importantly, however, using the zero shear rate viscosity *η*_0_ appeared to yield good convergence of the polymeric friction curves with the Newtonian baseline in the region prior to the onset of drag reduction, despite there being no apparent reason to anticipate this collapse *a priori* in TC flow, unlike in pressure-driven flows; the flow state immediately preceding featureless turbulence in our TC fixture is one of turbulent vortices^58^ and not a laminar shear flow (as is the case in pipe flows, where this initial preceding laminar state is responsible for the superposition of the dilute polymer measurements and the pure solvent curves^4^).

In the fully turbulent regime, the experimental friction curves for the dilute mucilage and PEO solutions diverge sharply away from the Newtonian baseline, as seen in Fig. 5a,b respectively; the upward deviation implies a decrease in the coefficient of friction *C*_*f*_ in comparison to the pure solvent, corresponding to the reduction in wall shear stress effected by the dissolved polymer chains at a given rotor speed. Analogous to the behaviour observed in pipe flows, the friction data for polymer solutions in the drag reducing regime could be described well by straight line fits on the Prandtl-von Kármán plot, given by

4 $$\sqrt{\frac{2}{{C}_{f}}}={M}_{p}\,\log \,{{\rm{Re}}}^{\ast }+{N}_{p},$$

which is similar in form to the pure solvent line, Eq. 3, albeit with a higher slope *M*_*p*_ > *M* that varies with the polymer additive used, and its concentration *c* in solution. Following Virk^65^, we define the slope increment *δ* = *M*_*p*_ − *M* as the change in slope with respect to the Newtonian baseline, and utilise this as a measure of the drag reducing efficacy of a given polymer solution. Insofar as the shear viscosity *η* appears only in the abscissa of the Prandtl-Kármán plot, a different choice for its value will only result in a horizontal shift of the curves in Fig. 5a,b; the slope *M*_*p*_, and by extension, the slope increment *δ* are thus independent of the specific choice of the shear viscosity used in calculating Re*.

In Fig. 5c, we plot the slope increment *δ* as a function of the normalised concentration *c*[*η*] for aqueous PEO and flax mucilage solutions. In the dilute limit, the slope increment *δ* for aqueous PEO solutions appears to scale with concentration as *δ* ~ (*c*[*η*])^1/2^; a similar square-root dependence on concentration has been reported for flexible drag-reducing chains in turbulent pipe flows^4,65^. At higher concentrations of dissolved PEO (*c*[*η*] ~ 0.1), *δ* reaches a maximum and subsequently decreases with concentration, indicating the onset of maximum drag reduction (MDR). In the case of flax mucilage, we observed a stronger dependence of the slope increment on the concentration, *δ* ~ (*c*[*η*])^0.58^, which we believe is a consequence of the presence of charged semiflexible chains comprising the acidic fraction of mucilage. It is also possible that the concentrations employed (even at 1:800 seed-to-water ratio) were not sufficiently dilute for the *δ* ~ *c*^1/2^ scaling behaviour to be applicable^65^.

### Practical considerations in engineering systems

The Prandtl-Kármán representation and the slope increment *δ*, as discussed above, serve as valuable tools in a semi-empirical approach to analysing and quantifying the drag reduction effect of polymer additives. Nonetheless, from a practical standpoint, the quantity that is often of immediate interest in engineering applications is the net decrease in fluid friction—either the wall shear stress in external flows, or the head loss per unit length in internal flows—gained in comparison to the unmodified (Newtonian) solvent under identical conditions of speed or flow rate. Accordingly, we define a percentage drag reduction Δ, given by

5 $$\Delta =\frac{{\tau }_{0}-\tau }{{\tau }_{0}}\times 100 \% $$

where *τ*_0_ and *τ* are, respectively, the frictional wall shear stresses measured in the Newtonian solvent (water in this case) and in the dilute polymer solution at the same rotor speed Ω, or equivalently, at the same Reynolds number Re_*s*_ = *ρ*(*r*_*i*_Ω)*w*/*η*_*s*_ calculated based on the pure solvent viscosity *η*_*s*_ (=*η*_*w*_). Defined this way, the percentage drag reduction Δ increases with increasing Reynolds number Re_*s*_ for both PEO and mucilage solutions, a direct consequence of the growing separation between the diverging polymer and solvent lines as seen in Fig. 5a,b. The presence of dissolved polymer chains, besides serving to suppress near-wall turbulence, also has the concomitant effect of increasing the shear viscosity *η* of the solution; this in turn enhances viscous dissipation in the flow, which counteracts the drag reduction effected by chain elasticity and thereby offsets the overall decrease in skin friction attained. Inasmuch as the viscoelastic suppression of near-wall turbulence is increasingly effective at larger Reynolds numbers (or higher turbulence intensities), there exists a threshold value of the Reynolds number Re_*s*_ above which the macromolecular disruption of turbulent momentum transport outweighs the increase in fluid viscosity, providing a net positive reduction in skin friction (or head loss) vis-á-vis the pure solvent in the absence of additives. This is evident in Fig. 5d, where the percentage drag reduction Δ is plotted as a function of the Reynolds number Re_*s*_ for an aqueous, 285 ppm solution of PEO, and a 1:400 extract (*c* = 460 ppm) of flax mucilage. Both solutions are drag-reducing at Reynolds numbers Re_*s*_ ≥ 26,000, but at lower speeds they generate more frictional drag on the rotor than pure water on account of their higher shear viscosity and the smaller turbulence intensity of the flow.

Referring again to Fig. 5d, we observe that the flax extract yields almost identical levels of drag reduction as the PEO solution throughout the drag-reducing regime (at the respective concentrations of each polymer mentioned above); the mucilage solution can thus potentially serve as an equally effective bio-sourced substitute for the synthetic PEO solution. The high cost of synthetic long chain polymers, and the logistic hurdles involved in transporting the requisite quantities of additives onboard, have been cited as the two principal roadblocks to the cost-effective deployment of polymers in marine and naval applications^2,12,23^. Costing studies in the past have generally concluded that marine drag reduction becomes economical at higher ship speeds, with increases in fuel prices, or with the availability of lower-cost drag-reducing polymers^23^. Industrial grade PEO marketed for large-scale use (for example, in paper manufacturing) is typically quite polydisperse, and contains a substantial fraction of low molar mass chains that are largely ineffectual in reducing frictional drag. To minimise the total dry volume of additives conveyed, and thereby render its onboard storage and handling practicable, the polymer stock should predominantly comprise high molar mass drag-reducing chains, which in turn entails expensive fractionation and processing steps that can significantly add to the net cost of production (for example, laboratory grade PEO of high molar mass and low polydispersity costs upwards of $300/kg). Assuming that high molar mass PEO can be produced at $50/kg (which we believe to be a fairly conservative estimate), the 285 ppm aqueous solution in Fig. 5d would incur a cost of $14/m^3^ for the polymer used in its preparation. By contrast, the price of unprocessed flax seeds was $0.37/kg as of January 2018^66^, which translates to a material cost of less than $1/m^3^ for the 1:400 mucilage extract in Fig. 5d that shows comparable drag reduction performance. This cost can be reduced even further if spent flax meal (post extraction of linseed oil) is substituted for fresh seeds as the source of mucilage^67^. A concurrent advantage of using seed mucilage as a drag reducing agent is the inherently low polydispersity of its high molar mass constituents (see Fig. 2), as is typically the case with many macromolecules of biological origin.

A key concern in the selection of viable drag reducing additives, especially for use in closed conduit flow applications such as pipelines, is the longevity of the drag reduction effect under sustained shear flow, which in turn is dictated by the resistance of the dissolved polymer chains to flow-induced degradation^68–70^. At high turbulence intensities, the elongational stresses on individual chains can grow sufficiently intense to cause scission of intrachain covalent bonds, leading to a progressive decline in the average chain length (or equivalently, the molar mass), and an attendant loss of drag reduction efficacy over time^71^. It has been reported that rigid polymers, such as those present in xanthan gum and okra mucilage, are more resistant to chain scission than flexible chains in turbulent flows; nevertheless, a decrease in percentage drag reduction still occurs with time, and is attributed to the de-aggregation of polymer chains under sustained shear^25,62^.

**Figure 6:**
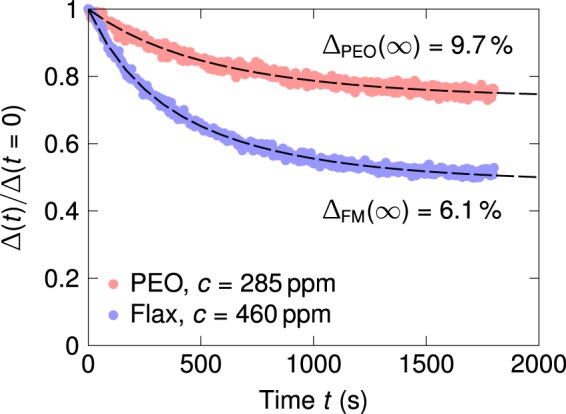
Decrease in the percentage drag reduction Δ(*t*) with time for the 285 ppm aqueous PEO solution and the 1:400 (*c* = 460 ppm) flax seed extract, under sustained shearing at Re_*s*_ = 62,000. The dashed lines show least squares fits of the Brostow model, Eq. 6, to the experimental data.

To study the effect of mechanical degradation, we subjected the 285 ppm aqueous PEO solution and the 1:400 flaxseed extract to prolonged shear flow in the TC fixture at a constant Reynolds number (Re_*s*_ = 62,000) and monitored the change in percentage drag reduction Δ(*t*) with time *t*. The experimental data is shown in Fig. 6. At *t* = 0, both solutions yielded comparable initial levels of drag reduction (Δ_PEO_(0) ≈ Δ_FM_(0) = 13%) at Re_*s*_ = 62,000. The diminution in drag reduction levels over time for the two solutions is well described using a least-squares regression to the Brostow equation^72–74^, given by

6 $$\frac{\Delta (t)}{\Delta (0)}=\frac{1}{1+W(1-{e}^{-bt})}$$

where *W* and *b* are fit parameters that denote, respectively, a representative number of breakage points per chain, and a characteristic time rate of degradation. The rates of degradation *b* (obtained from the fit) for the two solutions were comparable, with *b*_PEO_ ≈ *b*_FM_ = 1.4 × 10^−3^ s^−1^. In the limit of *t* → ∞, Eq. 6 gives Δ(∞) = Δ(0)/(1 + *W*), yielding a prediction for the asymptotic value of the percentage drag reduction expected at long times when the length of dissolved chains has been reduced sufficiently to render flow-induced scission processes inoperative^71^. For the two solutions in Fig. 6, we obtain Δ_PEO_(∞) = 9.7% and Δ_FM_(∞) = 6.1%; the terminal percentage drag reduction for the flax seed extract is smaller by about 37% as compared to the aqueous 2 MDa PEO solution. (For an analogous comparison with 5 MDa PEO which is more susceptible to mechanical degradation, see Fig. S6 in the Supplementary Material.)

**Figure 7:**
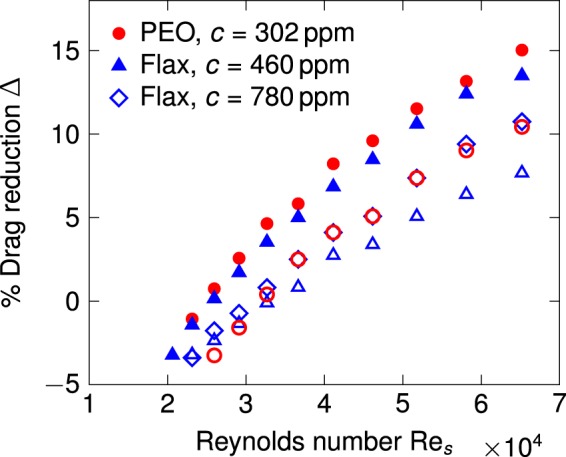
The percentage drag reduction Δ as a function of the Reynolds number Re_*s*_ for PEO and flax mucilage solutions in deionised water and synthetic seawater. The filled symbols denote experiments in deionised water, and the open symbols denote experiments in synthetic sea water.

Finally, an important practical consideration in employing polymer additives for marine drag reduction is the effect of salinity on the drag reduction efficacy of dissolved chains. The addition of salts, and the resulting decrease in the radius of gyration of chains in the solution due to charge screening, have been shown to adversely affect the drag-reducing ability of several water-soluble long chain polymers, including PEO. As expected, the effect is more severe in solutions of polyelectrolytes than in the case of neutral chains^75,76^. To study the effect of salinity on the percentage drag reduction, we performed flow measurements on solutions of PEO and flax mucilage in synthetic ocean water (ASTM D1141), which has a salinity of approximately 35 g/kg and an ionic strength equivalent to 0.6 M aqueous NaCl. As seen in Fig. 7, a 302 ppm solution of PEO and a 1:400 (*c* = 460 ppm) flax mucilage extract yield comparable drag reduction levels (filled symbols) in pure water free of dissolved salts. In synthetic seawater, both polymers display a loss of drag reduction efficacy (open symbols), with the mucilage solution experiencing a larger decrement in percentage drag reduction; this is expected on account of the acidic fraction in mucilage^28^, and is consistent with the sensitivity of the intrinsic viscosity [*η*] to the ionic strength discussed earlier in the text. We further observed that this drag reduction deficit relative to PEO (in seawater) could be compensated by increasing the seed-to-water weight ratio from 1:400 (*c* = 460 ppm) to 1:200 (*c* = 780 ppm) at the extraction stage (open diamonds in Fig. 7), which amounts to a roughly 70% increase in polysaccharide concentration in the final mucilage solution.

## Summary and Outlook

We performed experimental measurements of the efficacy of aqueous flaxseed mucilage as a drag reducing agent in turbulent flow, using a bespoke Taylor-Couette apparatus. Our results show that biopolysaccharides sourced from seed mucilage can deliver comparable performance as synthetic drag-reducing polymers, and potentially offer a cost advantage over the latter in prospective large-scale engineering applications. Synthetic and systems biology techniques have today enabled us to identify and alter plant and bacterial genomes involved in the biosynthesis of mucilage and extracellular polysaccharides^77–80^; these novel methods may in the future enable the selection and engineering of high-yield bacterial strains or plant cultivars that can support large-scale commercial extraction of biopolymers of even higher molar masses which are especially well-suited for turbulent drag reduction. With further advances in this direction, we believe that low cost, biodegradable, mucilage-based drag reducing agents may hold the potential to transform polymer drag reduction into a practical and cost-effective strategy for energy-efficient propulsion in real-life maritime applications.

## Methods

### Preparation of flax mucilage extracts

Aqueous mucilage solutions of different concentrations were prepared by combining raw (unground) brown flax seeds with deionised water at the desired weight ratio, and gently stirring the mixture at 80 °C for 30 min; for molar mass analysis, a seed to water weight ratio of 1:25 was employed during extraction, as per the optimal conditions reported by Ziolkovska^35^. The clear mucilage solution was separated from the seeds by filtering once through a coarse (grade 90) cotton mesh cloth, and then used directly in flow experiments. Gravimetric estimates of various mucilage components were obtained by lyophilising and carefully weighing the dry solid yield. The gel-like fraction was separated by centrifuging the aqueous mucilage extract at RCF = 10,000 × g for 30 min. The high molar mass polysaccharide fraction was isolated from the supernatant using a 100 kDa MWCO filter and subsequently analysed by size exclusion chromatography.

### Preparation of aqueous PEO

In the case of PEO, a concentrated 0.2% stock solution was first prepared by gently dispersing the requisite amount of dry PEO powder (nominal molar mass 2 × 10^6^ g/mol) in deionised water, and allowing the polymer to dissolve gradually over 2–3 days on a laboratory benchtop roller to ensure uniform, laminar mixing. The stock solution was later diluted with additional deionised water to the desired concentration.

### Size exclusion chromatography

SEC analysis of flax mucilage was performed using an Agilent PL-GPC 220 triple detection system equipped with a refractive index concentration detector, an inline viscometer, and a 650 nm wavelength dual-angle light scattering detector for absolute determination of molar mass. A 0.1 M aqueous solution of NaNO_3_ was used as the column eluent, with 0.02% sodium azide added as a bacteriostatic agent. The refractive index increment of mucilage polysaccharides, measured separately using a differential refractometer, was equal to d*n*/d*c* = 0.145 mL/g (40 °C, laser wavelength = 650 nm).

### Viscometry

The kinematic viscosities of dilute PEO and mucilage solutions were measured using a suspended-level glass capillary viscometer of the Ubbelöhde type (Size 0B, Cannon Instrument Company), with a viscometer constant equal to 5.21 × 10^−3^ mm^2^/s^2^ and a baseline efflux time of 172 s for pure water at 25 °C. Before each experiment, the viscometer (charged with the test solution) was allowed to adequately equilibriate with the surrounding water bath maintained at 25 °C. Measurements of the efflux time were performed manually using a handheld stopwatch (least count = 1 s), and were repeated for each solution until concordant readings were obtained. Intrinsic viscosities were determined by performing successive dilutions within the viscometer bulb, and simultaneously extrapolating the Huggins equation,

7 $$\frac{\eta -{\eta }_{s}}{{\eta }_{s}c}=[\eta ]+{k}_{H}{[\eta ]}^{2}c$$

and the Kraemer equation,

8 $$\frac{1}{c}\,\mathrm{ln}\,(\frac{\eta }{{\eta }_{s}})=[\eta ]-{k}_{K}{[\eta ]}^{2}c$$

to a common intercept at zero concentration^38^ (the individual plots are included in the Supplementary Material). Extrapolation in the linear regime was confirmed by verifying that *k*_*H*_ + *k*_*K*_ = 0.50 ± 0.03 in all cases. For intrinsic viscosity determination at zero ionic strength, the mucilage fraction was desalted and rinsed via multiple solvent exchange passes in a 100 kDa MWCO centrifugal filter. For intrinsic viscosity determination by isoionic dilution, the low molar mass fraction of the as-extracted mucilage solution (obtained as the filtrate in the first pass through the MWCO filter) was collected and used as the diluent in place of water.

### CaBER measurements

Measurements of the extensional relaxation time were performed on a CaBER apparatus consisting of two coaxial cylindrical plates (each of diameter 6 mm) that separate vertically and symmetrically away from a stationary midplane. The cylindrical fluid column bridging the gap between the plate faces was stretched rapidly from an initial height of 2 mm to a final height of 7 mm over a duration (or ‘strike’ time) of approximately 50 ms. The evolution of the thinning liquid filament was tracked concurrently using a laser micrometer as well as a high speed camera. Experiments were performed at room temperature, (23 ± 2) °C. At least three replicates were performed for each polymer solution. In the case of PEO, an aqueous solution of 0.67 g/mL sucrose (viscosity *η*_*s*_ = 0.0188 Pa s) was employed as the solvent. For flax mucilage, a more viscous solution of 0.83 g/mL sucrose in water (viscosity *η*_*s*_ = 0.012 Pa s) was used.

### Taylor-Couette flow experiments

Frictional drag measurements were performed using a bespoke Taylor-Couette (TC) flow apparatus, with its inner cylinder (rotor) coupled to a commercial rotational rheometer (AR-G2, TA Instruments) for precise speed control and torque measurements. The basic design and flow dimensions of the apparatus are described under the ‘Results’ section. The stator was made of transparent cast acrylic to provide visual access into the flow cell. The rotor was machined from aluminium 6061-T6 alloy, and its external cylindrical surface was polished to a mirror finish (root mean square roughness *R*_*q*_ ≤ 0.1 μm) to prevent roughness effects at the inner boundary layer. As designed, the TC apparatus could attain operating speeds as high as Ω = 140 rad/s (corresponding to a gap-based Reynolds number in excess of Re = 70,000), with the frictional shear stress reaching up to *τ* = 40 Pa at the rotor wall. The transition to fully turbulent (Newtonian) flow was determined to occur at a critical Reynolds number of Re_*c*_ ≃ 11,000^59,60^ in our particular TC geometry; the details of locating this transition point based on torque measurements in pure water are included in the Supplementary Material.

All flow measurements were performed at an ambient temperature of (23 ± 1) °C. In a typical experiment, the rotor speed was increased in discrete steps from Ω = 10 rad/s to Ω = 125 rad/s. At each step, the flow inside the gap was allowed to reach steady state, after which the frictional torque on the rotor was sampled and averaged over a period of 10 s. The standard deviation in torque values about the mean was less than 2% in all cases.

## Supplementary information

Supplementary Information

Supplementary Information

## Data Availability

All data generated or analysed during this study are either included in this article and its Supplementary Material, or available from the corresponding author on reasonable request.
