# Supplementary material for: Epidermal biopolysaccharides from plant seeds enable biodegradable turbulent drag reduction
Source: Sci Rep. 2019 Dec 4;9:18263. doi: 10.1038/s41598-019-54521-3 (PMC6892878; doi:10.1038/s41598-019-54521-3)
Supplement: Supplementary file 1 — Supplementary Information [file 41598_2019_54521_MOESM1_ESM.pdf]

Supplementary material for

Epidermal biopolysaccharides from plant seeds enable biodegradable turbulent drag reduction

Anoop Rajappan[1] and Gareth H. McKinley[1,*]

[1]Department of Mechanical Engineering, Massachusetts Institute of Technology, Cambridge, MA 02139, USA.
[*]gareth@mit.edu

1 Intrinsic viscosity measurements

The intrinsic viscosity $[\eta]$ of the aqueous PEO solution and flaxseed mucilage was measured experimentally by successive dilution inside an Ubbelöhde type suspended-level capillary viscometer (size 0B, Cannon Instrument Company), immersed in an isothermal water bath maintained at 25 °C. We adopted the conventional procedure[1] of computing both the reduced viscosity

$$\eta_{\text{red}} := \frac{\eta - \eta_s}{\eta_s c}, \tag{S1}$$

and the inherent viscosity

$$\eta_{\text{inh}} := \frac{1}{c} \ln\left(\frac{\eta}{\eta_s}\right), \tag{S2}$$

and simultaneously extrapolating η_{red} and η_{inh} to the limit of infinite dilution (η_s here is the viscosity of the solvent, and c the concentration of polymer in the solution). In the case of desalted mucilage fractions (see Fig. S1a), the reduced viscosity initially increased with dilution, a behaviour typically observed of polyelectrolyte chains in solution[2]. In this "chain expansion" regime, the solution viscosity showed good conformance to the empirical Fuoss equation[3,4] given by

$$\eta_{\text{red}} = \frac{A}{B + \sqrt{c}}, \tag{S3}$$

which is depicted by the dashed blue curve in Fig. S1a; the constants A and B above are fit parameters obtained from a least squares regression to the experimental viscosity data. Alternatively, Eq. S3 may be rearranged to yield a linear dependence of $1/\eta_{\text{red}}$ on $\sqrt{c}$, as observed in Fig. S1b for desalted flax mucilage.

With further dilution, the reduced viscosity of mucilage reaches a maximum and subsequently decreases, eventually displaying a linear dependence on concentration as in the case of neutral polymers[2]. In this linear region, we extrapolated the reduced and inherent viscosities concurrently to zero concentration, in accordance to the Huggins and Kraemer relations[1] which read, respectively,

$$\eta_{\text{red}} = [\eta] + k_{\text{H}}[\eta]^2 c + O\left(c^2\right) \tag{S4}$$

and

$$\eta_{\text{inh}} = [\eta] - k_{\text{K}}[\eta]^2 c + O\left(c^2\right). \tag{S5}$$

The common intercept of the two lines at $c = 0$ gives the intrinsic viscosity $[\eta]$ of desalted mucilage polysaccharides, as seen in Fig. S1c.

The polyelectrolyte-like behaviour of mucilage disappeared in the presence of dissolved salts[5,6], and the Fuoss regime was observed neither in the 0.5 M NaCl solution nor during isoionic dilution at the as-extracted ionic strength.

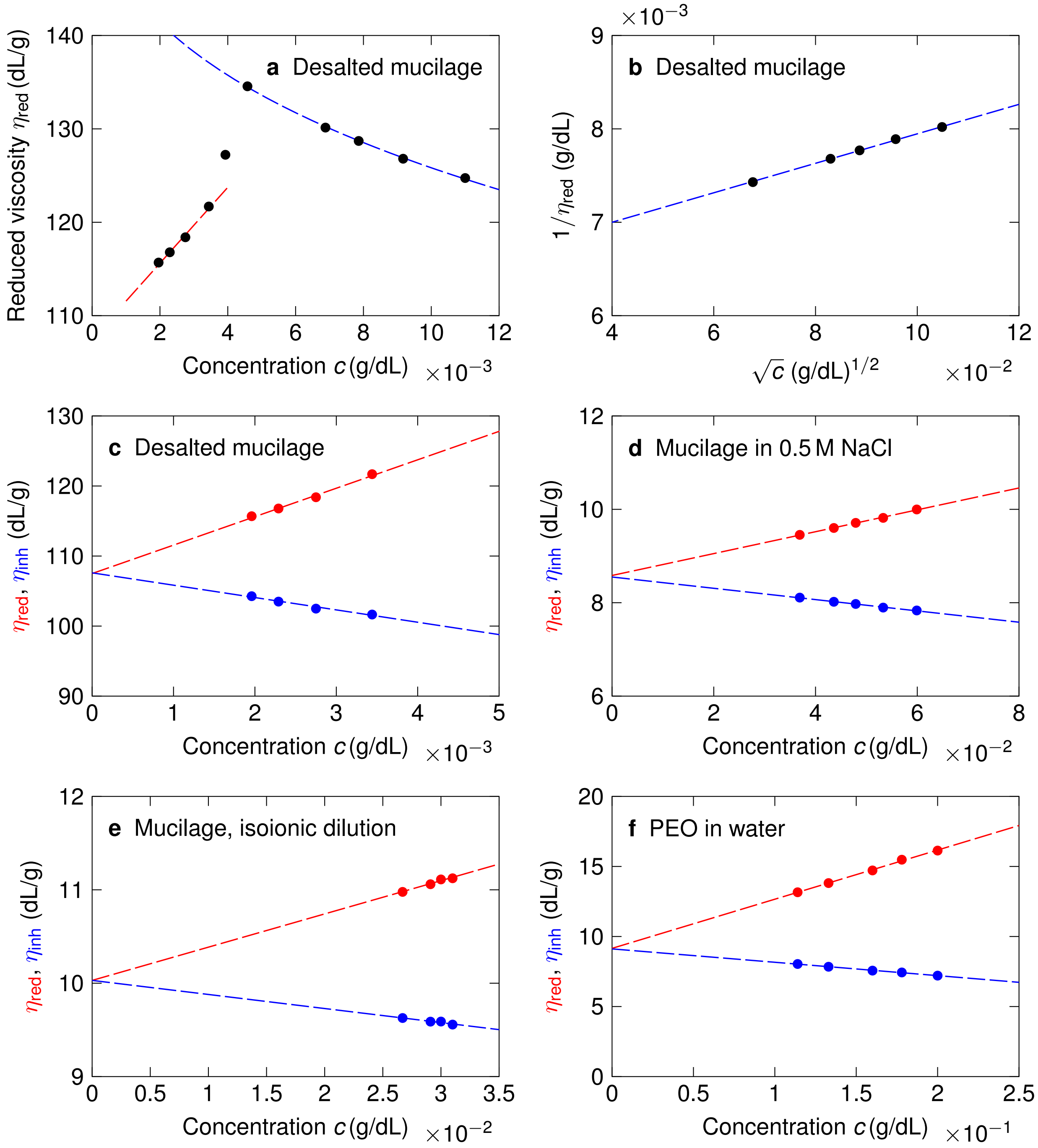

Figure S1. (a) Reduced viscosity η_{red} of purified and desalted aqueous flax mucilage, displaying polyelectrolyte-like behaviour. With progressive dilution, the reduced viscosity initially increases (dashed blue curve) consistent with the Fuoss equation[3,4], reaches a maximum, and subsequently decreases along the Huggins line (dashed red curve) at very low concentrations. (b) Fuoss plot of desalted flax mucilage, showing linear dependence of the inverse reduced viscosity $1/\eta_{red}$ on the square root of the concentration $\sqrt{c}$, for concentrations $c \geq 4.6 \times 10^{-3}$ g/dL. The data points and the blue dashed line are identical to those shown in panel (a). (c)–(f) Huggins-Kraemer plots constructed from experimental viscometry data, used to determine the intrinsic viscosity $[\eta]$ of (c) desalted flax mucilage, (d) flax mucilage in 0.5 M aqueous NaCl, (e) flax mucilage at the as-extracted ionic strength measured by isoionic dilution, and (f) aqueous solutions of 2 MDa PEO used in comparative studies with mucilage.

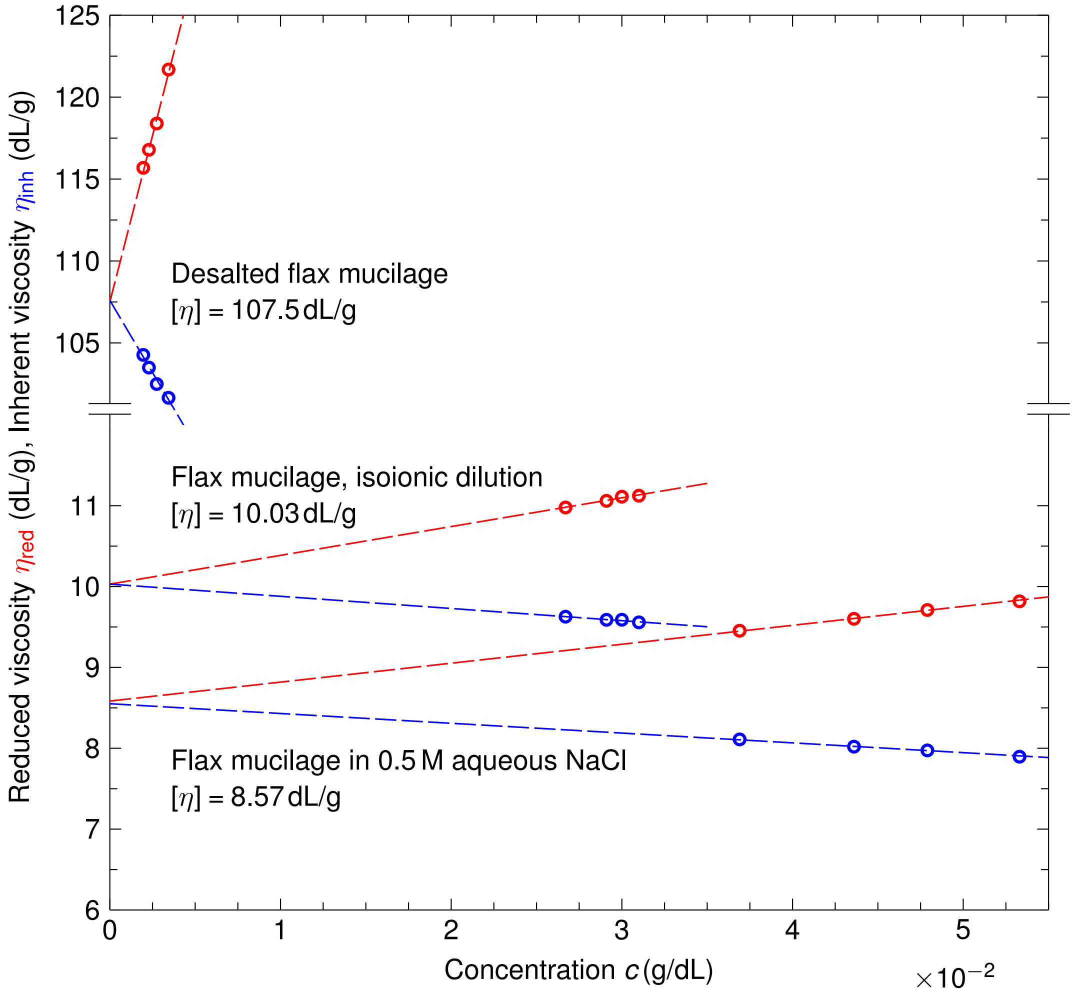

Figure S2. Intrinsic viscosity $[\eta]$ of aqueous flax mucilage measured at different ionic strengths. The data from Fig. S1, panels (c), (d) and (e) are reproduced here in a single composite figure for comparison.

In both cases, the Huggins regime was attained at larger concentrations than in the case of desalted mucilage, and the linear extrapolation procedure outlined above was directly employed to estimate the intrinsic viscosity $[\eta]$ of the dissolved polymer chain. The Huggins-Kraemer plots for flax mucilage at various salt concentrations, and for the neutral PEO chain used in comparative studies, are shown in Figs. S1c–f. In all cases, extrapolation in the linear regime was confirmed by verifying that $k_H + k_K = 0.50$ (within an error of ± 0.03), as required mathematically by the definitions of η_{red} and η_{inh} in Eqns. S1 and S2[1].

2 Transition to featureless turbulent flow in the TC apparatus

The bespoke TC fixture used in this study has a rotating inner cylinder coupled to the controlled-stress rheometer, and a transparent outer cylinder that remains stationary. The fluid angular momentum within the gap therefore decreases with increasing radius, and consequently, the primary azimuthal Couette flow is unstable to radial perturbations beyond a critical angular speed of the rotor. As the rotor speed is increased above this value, the flow passes through a number of states, and different kinds of secondary vortical flows appear and become stable at various Reynolds numbers[7]. At even higher speeds, these secondary flow structures become turbulent and eventually dissipate, until finally they cannot be detected any longer; at this stage, shear-driven featureless turbulent flow has been attained in the gap.[7,8].

Flow patterns inside the TC fixture were visualised with the help of a rheoscopic fluid, comprising 4 g/L of

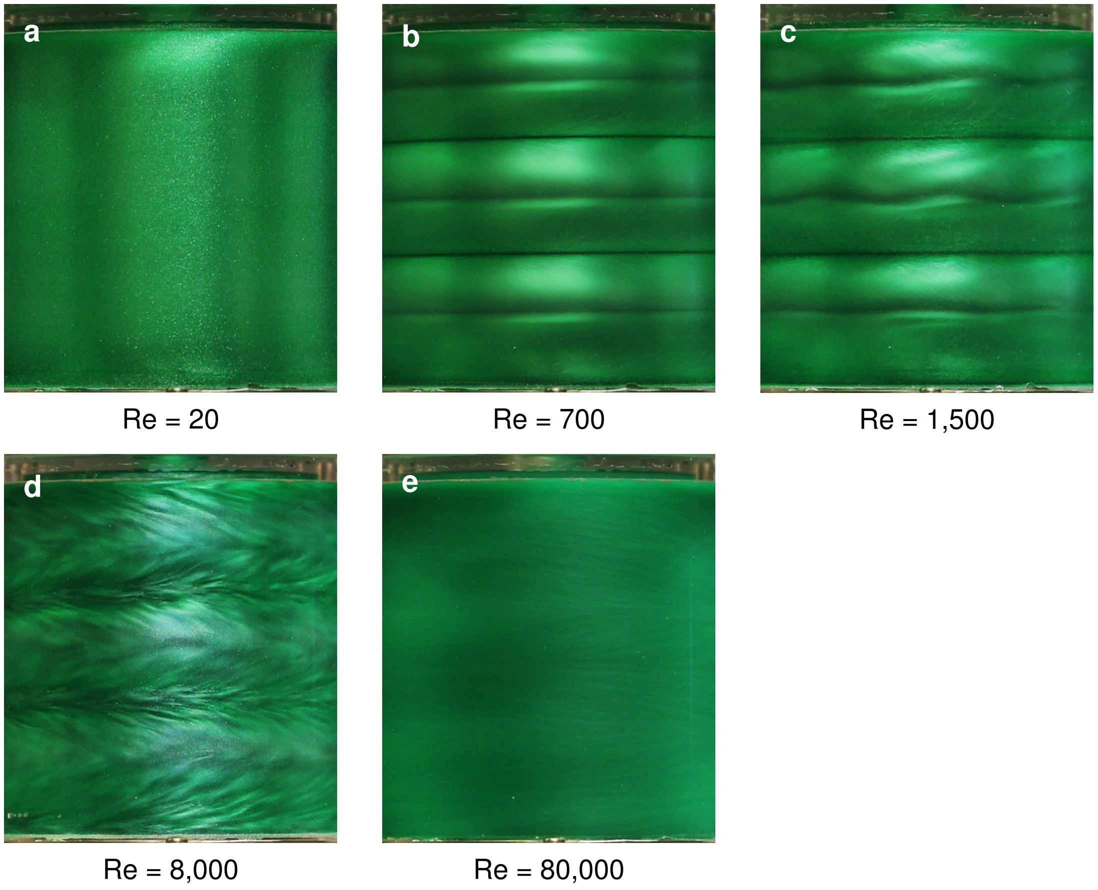

Figure S3. Representative flow patterns in the bespoke Taylor-Couette fixture observed at different Reynolds numbers Re during flow visualisation experiments. (a) laminar azimuthal Couette flow (AZI) at Re = 20; (b) Taylor vortex flow (TVF) with 3 vortex pairs at Re = 700; (c) vortex flow with wavy outflow boundaries (WOB) at Re = 1,500; (d) turbulent Taylor vortices (TTV) at Re = 8,000; and (e) featureless turbulence (TUR) at Re = 80,000.

finely powdered synthetic mica dispersed in a glycerol-water mixture of suitable viscosity; an 80 % glycerol solution was used for low Reynolds numbers up to 500, a 50 % solution was used for intermediate Reynolds numbers between 500 and 10,000, and pure deionized water was used at Reynolds numbers above 10,000. To further enhance visual contrast during imaging, the solution itself was dyed green using food coloring. Representative images of the flow patterns observed in the TC fixture at different Reynolds numbers are shown in Fig. S3.

To accurately locate the point of transition to featureless, fully turbulent flow inside gap, we further performed baseline friction measurements using Newtonian liquids of different viscosities; deionised water and two separate glycerol-water mixtures (57 % and 84 % glycerol in water, having viscosities $\eta = 1.30 \times 10^{-2}$ Pa s and $\eta = 1.18 \times 10^{-1}$ Pa s respectively) were used as working fluids to obtain skin friction data spanning six decades of the Reynolds number Re, in the range $10^{-1} \leq \mathrm{Re} \leq 10^5$. In each liquid, the steady state frictional torque $\mathscr{T}$ was measured over a range of discrete angular speeds Ω of the rotor. To compare friction curves obtained using fluids of different viscosities, we define a non-dimensional torque G given by[8]

$$G := \frac{\mathscr{T}}{\rho \nu^2 h}, \tag{S6}$$

where ρ and ν are respectively the density and the kinematic viscosity of the working fluid, and $h = 76.2$ mm is the rotor height. Fig. S4(a) shows the non-dimensional torque G as a function of the Reynolds number Re. Plotted this way, the three data sets collapse into a single Newtonian baseline curve for the TC fixture, and the data for different

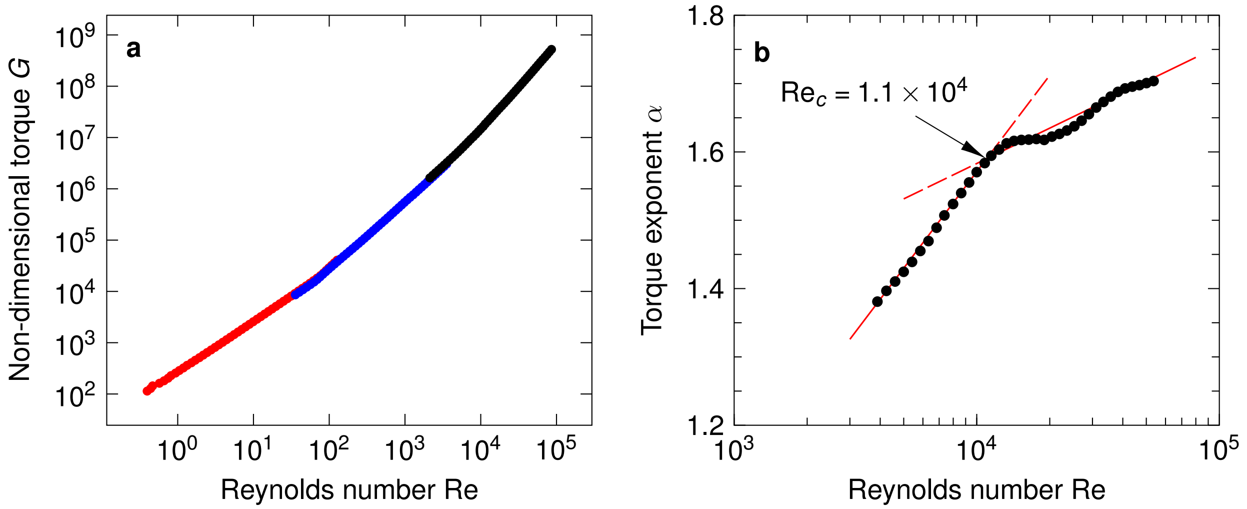

Figure S4. (a) Baseline skin friction curves for the bespoke Taylor-Couette fixture (radius ratio $\zeta = r_i/r_o = 0.75$, gap aspect ratio $\beta = h/w = 6.0$). Data points were obtained using water (black markers), 57 % glycerol in water (blue markers), and 84 % glycerol in water (red markers) as the working fluids, at different ranges of the Reynolds number Re. (b) The torque exponent α as a function of the Reynolds number Re. A change in slope is observed at $\mathrm{Re}_c = 1.1 \times 10^4$, indicating the point of transition to Newtonian, fully turbulent flow. The red lines denote piecewise linear fits to the torque exponent α before and after the transition point, as given by Eq. S9. (Reproduced from the Supplementary Material for Rajappan, A. *et al*, Influence of textural statistics on drag reduction by scalable, randomly rough superhydrophobic surfaces in turbulent flow, Phys. Fluids **31**, 042107 (2019), with the permission of AIP Publishing.)

liquids agree quite well in their regions of overlap. To determine the critical transition Reynolds number Re_c from this curve, we adopt the method used by Lathrop, Fineberg and Swinney[8], and assume a power law scaling for the frictional torque at large Reynolds numbers, of the form

$$G \sim \mathrm{Re}^{\alpha} \tag{S7}$$

and compute the torque exponent α, given by

$$\alpha = \frac{\mathrm{d}\log G}{\mathrm{d}\log \mathrm{Re}}. \tag{S8}$$

The data set for large Reynolds numbers, obtained using water as the working fluid, consists of discrete torque measurements spaced at uniform log intervals of $\Delta(\log_{10} G) = 0.05$, spanning the range of Reynolds numbers $2.1 \times 10^3 \leq \mathrm{Re} \leq 8.6 \times 10^4$. Since direct numerical differentiation accentuates noise in the data, the value of α was instead calculated using a sliding linear least squares fit over 15 adjacent data points (equivalent to a log interval of $\Delta(\log_{10} G) = 0.70$). The exponent α is plotted as a function of the Reynolds number in Fig. S4(b). The torque exponent α increases monotonically with the Reynolds number Re, suggesting that there is no single power law scaling that holds uniformly throughout this range of Reynolds numbers. However, a sharp discontinuity in the slope of the curve is observed at $\mathrm{Re} = 1.1 \times 10^4$, indicating a change in the dependence of α on the Reynolds number Re (and by extension, the scaling of G with Re). Following Lathrop et al.[8], we identify $\mathrm{Re}_c = 1.1 \times 10^4$ as the critical Reynolds number at which the (Newtonian) wall-driven flow inside the gap of our bespoke TC fixture transitions to shear-driven, featureless turbulence.

An empirical equation for α may be derived by means of a piecewise linear regression to the data in the vicinity

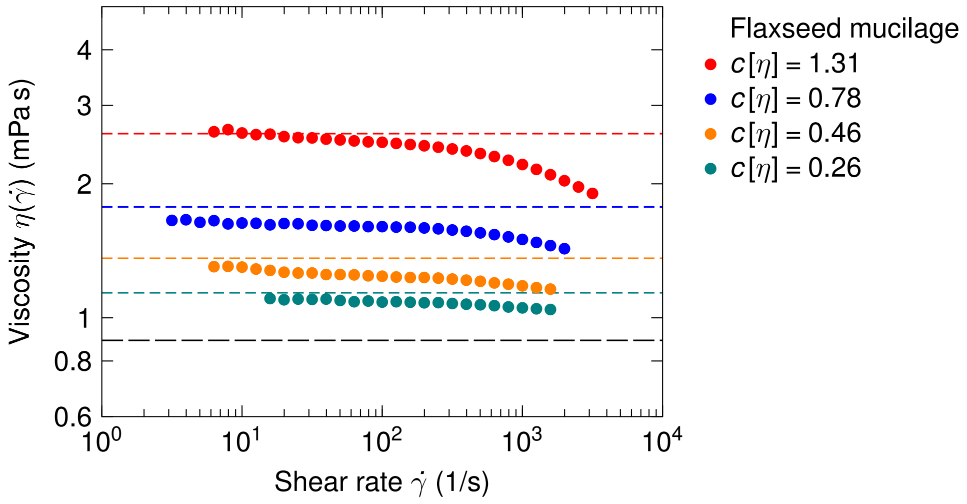

Figure S5. Shear viscosity of dilute flax mucilage solutions of various concentrations used in TC flow experiments. The critical overlap concentration for the mucilage is approximately $c^* = 1/[\eta] = 1.00\,\mathrm{g/L}$. The data markers denote the viscosity $\eta(\dot{\gamma})$, as a function of the shear rate $\dot{\gamma}$, measured using the AR-G2 rheometer, and the broken lines represent the viscosity obtained independently using an Ubbelöhde glass capillary viscometer. The black dashed line at the bottom denotes the viscosity of pure deionised water ($\eta_w = 8.9 \times 10^{-4}\,\mathrm{Pa\,s}$), representing an absolute lower limit on the viscosity of the solutions. All viscosity data were measured at a temperature of 25 °C.

of the transition point:

$$\alpha = \begin{cases} 1.59 + 0.469 \log_{10}(\mathrm{Re}/\mathrm{Re}_c) & \mathrm{Re} \le \mathrm{Re}_c \\ 1.59 + 0.172 \log_{10}(\mathrm{Re}/\mathrm{Re}_c) & \mathrm{Re} > \mathrm{Re}_c \end{cases} \tag{S9}$$

At the transition point, the critical exponent $\alpha = 1.59$, which is close (but not exactly equal) to the marginal stability exponent[8] of $\alpha = 5/3$. The value of $\mathrm{Re}_c = 1.1 \times 10^4$ obtained above for our TC fixture, which has a radius ratio of $\eta = 0.75$, compares well to the value of $\mathrm{Re}_c = 1.3 \times 10^4$ obtained by Lathrop et al. for their TC apparatus, which had a radius ratio of $\eta = 0.72$[8].

3 Shear viscosity of dilute flax mucilage solutions

We utilized a commercial controlled-stress rotational rheometer (AR-G2, TA Instruments) to measure the steady shear viscosity $\eta(\dot{\gamma})$ of the dilute flax mucilage solutions employed in drag reduction tests, as function of the shear rate $\dot{\gamma}$. Measurements were performed at 25 °C using a standard double-gap concentric cylinder geometry, and the results are shown in Fig. S5. At the mucilage concentrations used, no appreciable shear thinning was observed at shear rates $\dot{\gamma} \le 10^3\,\mathrm{s}^{-1}$. Furthermore, the viscosity data at low shear rates was in good agreement with the viscosity η_0 obtained from manual efflux time measurements in an Ubbelöhde type suspended-level glass capillary viscometer (size 0B, Cannon Instrument Company). This latter value of the solution viscosity was therefore used to compute the shear Reynolds number Re^* used in the Prandtl-von Kármán plots, Fig. 5, in the main text.

4 Mechanical degradation: comparing flax mucilage with 5 MDa PEO

We subjected a 10 ppm aqueous solution of 5 MDa PEO, and a 1:800 flaxseed extract ($c = 260\,\mathrm{ppm}$), to prolonged shear flow in the TC fixture at a constant Reynolds number ($\mathrm{Re}_s = 59{,}400$) and monitored the change in percentage drag reduction $\Delta(t)$ with time t. The experimental data is shown in Fig. S6. At $t = 0$, both solutions yielded comparable initial levels of drag reduction ($\Delta_{\mathrm{PEO}}(0) = 21\,\%$, and $\Delta_{\mathrm{FM}}(0) = 20\,\%$) at $\mathrm{Re}_s = 59{,}400$. The decline in the drag reduction performance over time for the two solutions was modelled using a least-squares fit to the Brostow

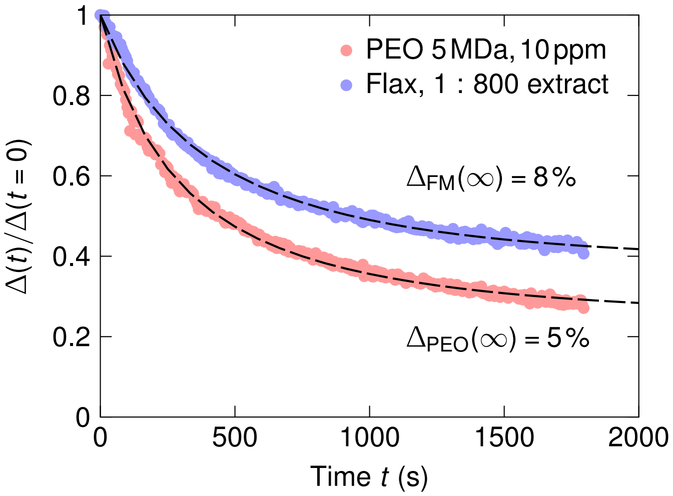

Figure S6. Decrease in the percentage drag reduction $\Delta(t)$ with time for a 10 ppm solution of 5 MDa PEO and a 1:800 flax seed extract, under sustained shearing at $\mathrm{Re}_s = 59{,}400$. The two solutions gave comparable initial levels of drag reduction at this Reynolds number, with $\Delta_{\mathrm{PEO}}(0) = 21\,\%$, and $\Delta_{\mathrm{FM}}(0) = 20\,\%$. The dashed lines show least squares fits of the Brostow model, Eq. S10, to the experimental data.

equation[9–11], given by

$$\frac{\Delta(t)}{\Delta(0)} = \frac{1}{1 + W\left(1 - e^{-bt}\right)} \tag{S10}$$

where W and b are fit parameters that denote, respectively, a representative number of breakage points per chain, and a characteristic time rate of degradation. The rates of degradation b (obtained from the fit) for the two solutions were comparable, with $b_{\mathrm{PEO}} = 9.3 \times 10^{-4}\,\mathrm{s}^{-1}$ for the PEO solution, and $b_{\mathrm{FM}} = 1.1 \times 10^{-3}\,\mathrm{s}^{-1}$ for the mucilage extract. As $t \to \infty$, Eq. S10 predicts $\Delta(\infty) = \Delta(0)/(1+W)$, yielding an asymptotic value of the terminal percentage drag reduction to be expected at long times when the dissolved chains have been degraded enough to render further flow-induced scission inoperative[12]. Again, we obtained comparable values of asymptotic drag reduction for the two solutions, with $\Delta_{\mathrm{PEO}}(\infty) = 5\,\%$ for the PEO solution, and $\Delta_{\mathrm{FM}}(\infty) = 8\,\%$ for the flax extract. The mucilage solution thus performs equally well as aqueous 5 MDa PEO in this case, showing a similar longevity and resistance to degradation under prolonged exposure to turbulent shear flow.

5 Supplementary tables

Table S1 lists the concentrations c and the extensional relaxation times λ for all aqueous PEO and flax mucilage solutions used in drag reduction experiments in this work. For flax mucilage, the value of the intrinsic viscosity reported is the one obtained by isoionic dilution, and the relaxation times λ were estimated from the power law fit in Fig. 3d. For 5 MDa PEO, the Zimm relaxation time λ_Z was computed using Eq. 2 in the main text.

6 Supplementary movies

Movie S1 The as-extracted aqueous flax mucilage solution displaying tubeless siphoning, on account of the nonlinear extensional viscoelasticity imparted by dissolved long chain polysaccharides. The movie shows the mucilage solution from the lower reservoir being drawn upwards into a syringe that is raised and held above the free surface, via a "tubeless" liquid bridge supported by elastic stresses induced by the molecular elongation of the dissolved polysaccharides. The mucilage solution used in this demonstration was extracted at a seed-to-water ratio of 1:8, and a red food colouring was added to the solution to enhance visualisation. The solutions used in drag reduction experiments were significantly more dilute, and were extracted at seed-to-water ratios ranging between 1:100 and 1:800.

Table S1. Concentrations and extensional relaxation times of aqueous PEO and flax mucilage solutions used in drag reduction experiments. For the mucilage extracts, the ratio by mass of flax seeds to water employed during hot water extraction is also reported.

Polymer		Concentration c [ppm]	Normalised concentration $c[\eta]$ [–]	Relaxation time λ [ms]
	1:800	260	0.26	0.03
Flax mucilage	1:400	460	0.46	0.05
$[\eta] = 10.03\,\mathrm{dL/g}$	1:200	780	0.78	0.10
	1:100	1310	1.31	0.18
		285	0.26	2.74
PEO 2 MDa		504	0.46	3.74
$[\eta] = 9.13\,\mathrm{dL/g}$		865	0.79	4.49
		1460	1.33	5.67
PEO 5 MDa $[\eta] = 20.2\,\mathrm{dL/g}$		10	0.02	$\lambda_Z = 3.65\,\mathrm{ms}$

References

1. Pamies, R., Cifre, J. G. H., del Carmen López Martínez, M. & de la Torre, J. G. Determination of intrinsic viscosities of macromolecules and nanoparticles. Comparison of single-point and dilution procedures. *Colloid Polym. Sci.* **286**, 1223–1231 (2008).
2. Zheng, K., Chen, K., Ren, W., Yang, J. & Zhao, J. Counterion cloud expansion of a polyelectrolyte by dilution. *Macromolecules* **51**, 4444–4450 (2018).
3. Fuoss, R. M. Viscosity function for polyelectrolytes. *J. Polym. Sci.* **3**, 603–604 (1948).
4. Tanford, C. *Physical Chemistry of Macromolecules* (John Wiley and Sons, Inc., 1961).
5. Fuoss, R. M. & Strauss, U. P. Electrostatic interaction of polyelectrolytes and simple electrolytes. *J. Polym. Sci.* **3**, 602–603 (1948).
6. Muthukumar, M. 50th anniversary perspective: A perspective on polyelectrolyte solutions. *Macromolecules* **50**, 9528–9560 (2017).
7. Andereck, C. D., Liu, S. S. & Swinney, H. L. Flow regimes in a circular Couette system with independently rotating cylinders. *J. Fluid Mech.* **164**, 155–183 (1986).
8. Lathrop, D. P., Fineberg, J. & Swinney, H. L. Transition to shear-driven turbulence in Couette-Taylor flow. *Phys. Rev. A* **46**, 6390–6405 (1992).
9. Brostow, W. Drag reduction and mechanical degradation in polymer solutions in flow. *Polymer* **24**, 631–638 (1983).
10. Brostow, W., Ertepinar, H. & Singh, R. P. Flow of dilute polymer solutions: Chain conformations and degradation of drag reducers. *Macromolecules* **23**, 5109–5118 (1990).
11. Kim, C. A., Kim, J. T., Lee, K., Choi, H. J. & Jhon, M. S. Mechanical degradation of dilute polymer solutions under turbulent flow. *Polymer* **41**, 7611–7615 (2000).
12. Vanapalli, S. A., Islam, M. T. & Solomon, M. J. Scission-induced bounds on maximum polymer drag reduction in turbulent flow. *Phys. Fluids* **17**, 095108 (2005).
